# Time to minimal disease activity in relation to quality of life, productivity, and radiographic damage 1 year after diagnosis in psoriatic arthritis

**DOI:** 10.1186/s13075-019-1811-4

**Published:** 2019-01-16

**Authors:** Kim Wervers, Jolanda J. Luime, Ilja Tchetverikov, Andreas H. Gerards, Marc R. Kok, Cathelijne W. Y. Appels, Wiebo L. van der Graaff, Johannes H. L. M. van Groenendael, Lindy-Anne Korswagen, Josien J. Veris-van Dieren, Johanna M. W. Hazes, Marijn Vis, on behalf of Cicero

**Affiliations:** 1 Department of Rheumatology, Erasmus University Medical Center, Wytemaweg 80, 3015 CN Rotterdam, The Netherlands; 2 Department of Rheumatology, Albert Schweitzer Hospital, Dordrecht, The Netherlands; 3 Department of Rheumatology, Vlietland Hospital, Schiedam, The Netherlands; 4 Department of Rheumatology, Maasstad Hospital, Rotterdam, The Netherlands; 5 Department of Rheumatology, Amphia Hospital, Breda, The Netherlands; 6 Department of Rheumatology, Rivas Hospital, Gorinchem, The Netherlands; 7 Department of Rheumatology, Reumazorg Zuid West Nederland, Roosendaal, The Netherlands; 8 Department of Rheumatology, Sint Franciscus Gasthuis, Rotterdam, The Netherlands

**Keywords:** Psoriatic arthritis, Minimal disease activity, Health-related quality of life, Productivity, Composite measure

## Abstract

**Background:**

In a cohort of patients with newly diagnosed psoriatic arthritis (PsA) who received usual care, we investigated the impact of time elapsed to minimal disease activity (MDA) on health-related quality of life (HRQoL), work productivity, and radiographic damage throughout the first year after diagnosis.

**Methods:**

Data collected in the Dutch southwest early PsA cohort (DEPAR) study were analyzed. These three-monthly data encompassed disease activity, HRQOL was measured with the Short Form 36 (SF36) Physical Component Scale (SF36-PCS) and Mental Component Scale, and productivity was measured with the Productivity Cost Questionnaire. Radiographic damage was scored at baseline and at 12 months with the PsA-modified Sharp/van der Heijde score. Patients were classified by time to MDA as in early (within 3 months), late (at 6–12 months), and never MDA in the first year.

**Results:**

We included 296 patients who had had their 1-year outpatient visit (mean age 51 years, 53% male). Ninety-six (32%) were classified as early MDA, 78 (26%) as late MDA, and 98 (33%) as never MDA. Data of 24 patients (8%) were missing. SF36-PCS and productivity scores improved after gaining MDA, but remained low in never MDA patients. At 1 year, SF36-PCS and productivity scores were similar in early and late MDA patients. Radiographic progression rate was low and similar in all groups.

**Conclusion:**

Gaining MDA was associated with considerable improvement in HRQoL and functioning, irrespective of time to first MDA. In the one third of patients not in MDA in the first year, the disease had a substantial health impact.

## Background

Psoriatic arthritis (PsA) is a chronic inflammatory disease presenting with arthritis, enthesitis, spondylitis, dactylitis, and psoriasis [1]. As in other rheumatic diseases, chronic inflammation leads to progressive joint damage, increased disability, and reduced life expectancy [2–4]. Treatment with disease-modifying antirheumatic drugs (DMARDs) can alleviate this inflammation and prevent these complications [5, 6]. Better clinical, functional, and structural outcomes have been achieved with the use of treat-to-target strategies in patients with rheumatoid arthritis [7]. This implies that treatment is intensified until a target of low disease activity is reached. This strategy has been recommended in the treatment of PsA as well [8], although as yet there is no consensus of which target to use. Minimal disease activity (MDA) is one of the proposed treatment targets in PsA.

MDA is assumed to have been reached when at least five out of seven PsA remission criteria are met [9]. In previous cross-sectional studies, a state of MDA was strongly related with better functioning and health-related quality of life (HRQoL) [10–12]. Longitudinal studies confirming these findings are lacking. We need more information on how the state of MDA is related to long-term improvement in disability and whether achieving MDA early after the diagnosis results in better outcomes than achieving MDA at a later stage.

We investigated the impact of time elapsed from diagnosis to MDA on HRQoL, productivity, and radiographic damage at 1-year follow-up in a cohort of patients newly diagnosed with PsA and receiving usual care. We established if patients had reached MDA early, late, or never in the first year after diagnosis and how their HRQoL, work productivity, and radiographic damage was throughout the first year after diagnosis.

## Methods

### Patients and setting

We used data collected in the Dutch southwest Early Psoriatic Arthritis cohoRt (DEPAR) study, of which details are described elsewhere [11]. Patients with a new diagnosis of PsA are eligible to participate if they have not yet received treatment with DMARDs for PsA. Written informed consent was obtained from all participants according to the Declaration of Helsinki. The study was approved by the medical research ethics committee of the Erasmus University Medical Center Rotterdam, the Netherlands (MEC-2012-549). We analyzed data collected between August 2013 and June 2017 and excluded patients with baseline visits after June 2016.

### Data collection

In the first year, data are collected every 3 months. During the study visits, trained research nurses collect clinical data, including swollen and tender joint count (resp. SJC 66 and TJC 68 joints), enthesitis at clinical examination (Leeds Enthesitis Index, LEI [13]), and psoriasis (Psoriasis Area and Severity Index, PASI [14]). Patients complete multiple questionnaires shortly before or after their visits. For this analysis, we used data on the Short Form 36 (SF36, [15]), the Health Assessment Questionnaire (HAQ, [16]), the Productivity Cost Questionnaire (PCQ, [17]), and patient-reported visual analog scale (VAS) scores for global and pain.

### Minimal disease activity

MDA state was determined at each visit within the first year. A state of MDA was assumed to have been reached if five of seven MDA criteria were met: SJC ≤ 1, TJC ≤ 1, LEI ≤ 1, PASI ≤ 1, global VAS ≤ 20 mm, pain VAS ≤ 15 mm, HAQ ≤ 0.5 [9]. In case of missing data of some criteria, MDA status was ascertained irrespective of the missing information (e.g., fulfilling five criteria and missing two criteria). If missing information could alter the MDA status (e.g., fulfilling four criteria and missing two), we considered MDA status missing. If MDA status before and after a missing visit was equal (i.e., both MDA or both no MDA), we assumed that MDA status at the missing visit was equal to that state. Achieving MDA was classified as early if it had been achieved at baseline or at 3 months, as late if it was first achieved after 3 months but within the first year, and as never if it was not achieved in the first year. In addition, achieving MDA was classified as sustained if patients remained in MDA until their 1-year follow-up visit.

### Outcomes

Patients self-reported HRQoL with the SF36 Physical Component Scale (PCS) and Mental Component Scale (MCS). Work productivity was assessed with the PCQ, relating to work and productivity in the 4 weeks preceding the study visit. We determined employment status, absence, working hours, and productivity loss at work and at home throughout the first year. The actual number of productivity hours per week was calculated by subtracting hours of absence and productivity loss at work in hours from the total number of self-reported working hours.

Radiographs of the hand and feet obtained at baseline and after 1 year were scored twice, by three trained assessors separately. The assessors were blinded to the patient’s identity and clinical data and scored the images in chronological order. According to the PsA-modified Sharp/van der Heijde score (PsA-SHS), erosions and narrowing of hand and feet joints were scored with a maximum of 320 for erosions and 208 for joint space narrowing [18]. Differences in absolute score above 2 and differences in progression were discussed by the two assessors. Regarding the inter-rater reliability, the kappa statistics was 0.79 with absolute agreement of 99%. For each patient, the mean of two scores was calculated. The smallest detectable difference (SDD) was 0.27. Progression was defined as a mean increase in PsA-SHS in the first year exceeding the SDD.

### Statistical analysis

Differences in characteristics between MDA groups were tested with ANOVA tests and subsequent *t*-tests for continuous data and chi-squared tests for categorical data. The effect of time to MDA on outcomes after 1 year was analyzed with multiple linear regression analysis with outcomes SF36-PCS, SF36-MCS, and productivity. The association of time to MDA (early vs. late, early vs. never and late vs. never) with the outcome was corrected for baseline score SF36 or productivity score, age, gender, symptom duration, DMARD use, erosive disease at baseline, and baseline disease activity (baseline SJC, TJC, LEI, PASI, VAS global, VAS pain, and HAQ). In the subset of patients using DMARDs in the first year, the same analysis was performed with the added variables time to start and duration of DMARD therapy. Linear mixed effects models were used to compare progression of outcomes between the three groups and to account for missing data. Baseline outcome score, baseline disease activity and erosive disease, age, gender, symptom duration, DMARD use, time, MDA group, and interaction between time and MDA group were included in the fixed-effects part. Random intercepts were included in the random-effects part; an optimal random-effects structure was chosen based on the Akaike information criterion. Analyses were performed in STATA 15.1 and R-3.4.2.

## Results

In July 2017, 296 patients had had their 1-year visit and 268 (92%) could be assigned an MDA category. The mean age of the latter was 51.2 years (standard deviation (SD) 14), 142 were male (53%), and the median self-reported symptom duration was 1.0 years (interquartile range (IQR) 0.4–2.7, Table 1). Ninety-four (35%) had achieved MDA within 3 months (early MDA; 40 already in MDA at baseline), 77 (29%) between 6 and 12 months (late MDA), and 97 (36%) were never in MDA during the first year (never MDA). MDA state was sustained until 1 year by 43 (48%) early MDA patients and 46 (52%) late MDA patients. Patients early in MDA were significantly more often in MDA than patients late in MDA (of 5 visits: mean 3.6 vs. 1.8, *p* < 0.01).

**Table 1:** Patient characteristics of MDA groups and of total study population

	Early MDA (*n* = 94)	Late MDA (*n* = 77)	Never MDA (*n* = 97)
Age	50.1 ± 14	50.4 ± 13	52.8 ± 14
Male	57 (61)	41 (53)	44 (45)
Symptom duration in years	0.9 (0.2–2.4)	0.8 (0.3–1.5)	1.6 (0.4–4.1)
Disease activity at baseline			
Swollen joint count (66)	1 (1–3)	3 (1–4)	2 (0–6)^$^
Tender joint count (68)	1 (0–3)	3 (2–7)*	5 (3–12)^$^
LEI	0 (0–0)	0 (0–1)*	1 (0–2)^$^
HAQ	0.13 (0.00–0.63)	0.63 (0.38–0.89)*	0.88 (0.63–1.38)^$^
PASI	1.5 (0.4–3.8)	2.3 (0.3–4.1)	3 (1.2–6.1)^$^
VAS global	21 (9–37)	46 (24–66)*	55 (40–70)^$^
VAS pain	17 (9–44)	47 (30–63)*	56 (44–74)^$^
Medication in first year			
Any DMARD	62 (66)	63 (82)*	85 (88)^$^
Methotrexate	57 (61)	61 (79)*	79 (81)^$^
Sulfasalazine	8 (9)	14 (18)	23 (24)^$^
Hydroxychloroquine	4 (4)	11 (14)	8 (8)
Leflunomide	8 (9)	8 (10)	17 (18)
Prednisone (oral)	13 (14)	14 (18)	18 (19)
Prednisone (intramuscular injection)	11 (12)	21 (27)*	34 (35)^$^
Prednisone (intra-articular injection)	29 (31)	27 (35)	15 (15)^$^
Biological	5 (5)	12 (16)*	16 (16)

Results shown as mean ± standard deviation, *n* (%) or median (interquartile range). *Early vs. Late MDA and ^$^MDA vs. Never MDA *p* < 0.05 of *t*-test (continuous data) or chi-squared (categorical data). *MDA* minimal disease activity, *Early MDA* MDA within 3 months, *Late MDA* MDA between 3 and 12, *Never MDA* no MDA within first year. *LEI* Leeds Enthesitis Index, *HAQ* Health Assessment Questionnaire, *PASI* Psoriasis Area and Severity Index, *VAS* visual analogue scale, *DMARD* disease-modifying antirheumatic drug

Age, gender, and symptom duration did not differ significantly between groups (Table 1). Patients never in MDA had significantly higher SJC, TJC, LEI, HAQ, PASI, and VAS scores at baseline than patients in MDA (both early and late). Patients late in MDA had significantly higher disease activity than patients early in MDA on all domains but SJC and PASI. In the first year, DMARDs were prescribed to 210 patients (78%): methotrexate to 197 patients (74%) and biological therapy to 33 patients (12%).

### Quality of life

**Fig. 1:**
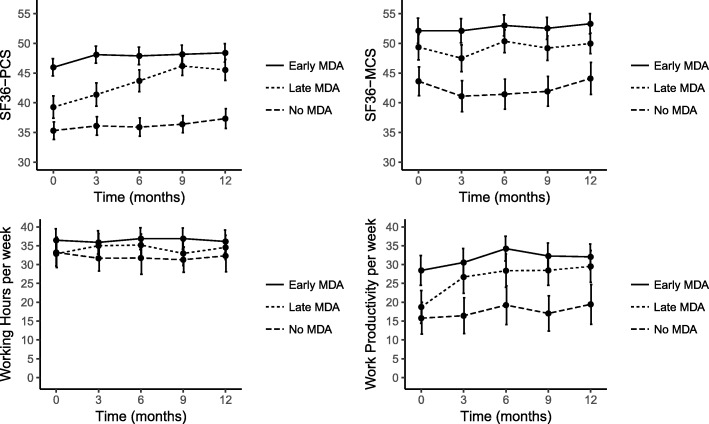
HRQoL and productivity outcomes in the first year per MDA group. SF36: Short Form 36, PCS: Physical Component Scale, MCS: Mental Component Scale, MDA: minimal disease activity. Results shown as mean and 95% confidence interval

The evolvement of the mean SF36-PCS and SF36-MCS scores per group is shown in Fig. 1. The mean SF36-PSC score at 1 year was similar in the early and late MDA groups and was better than that in the never MDA group. Linear regression analysis of the SF36-PCS score at 1 year, correcting for baseline score, gender, age, symptom duration, DMARD use, erosive disease at baseline, and baseline disease activity, showed that SF36-PSC scores were significantly lower in the never MDA group than in the early MDA group (*β* − 6.22, 95% CI − 9.92, − 2.51) and the late MDA group (*β* − 7.86, 95% CI − 11.52, − 4.21, Table 2). At baseline, SF36-PCS scores in the early MDA group were higher than those in the late MDA group and the never MDA group and had increased earlier than in the late MDA group. Linear mixed model analysis confirmed a difference in evolution over time between the three groups (likelihood ratio test (LRT) of interaction between time and group: 34.5, *p* < 0.0001). Results in the subgroup of patients using DMARDs were similar in analyses correcting for duration of DMARD therapy and time to start DMARD therapy. The SF36-MCS scores significantly differed between the three groups at baseline and remained fairly stable over time, which was confirmed by a non-significant interaction term of time and MDA in the linear mixed model analysis (LRT 1.73, *p* = 0.42). At 1 year, the SF36-MCS scores were significantly higher in the early MDA group than in the never MDA group (*β* − 6.11, 95% CI − 9.51, − 2.70, Table 2).

**Table 2:** Linear regression models outcomes after 1 year

	*β* (95% CI)
SF36-PCS	
Early vs. Late MDA	1.64 (−1.63, 4.91)
Early vs. Never MDA	−6.22 (−9.92, −2.51)
Late vs. Never MDA	−7.86 (− 11.52, −4.21)
SF36-MCS	
Early vs. Late MDA	−2.83 (−6.14, 0.06)
Early vs. Never MDA	−6.11 (−9.51, − 2.70)
Late vs. Never MDA	−3.07 (− 6.16, 0.02)
Hours productivity loss	
Early vs. Late MDA	0.78 (−5.63, 7.20)
Early vs. Never MDA	−6.59 (−14.55, 1.37)
Late vs. Never MDA	−7.38 (−14.38, −0.38)

*Early MDA* MDA within 3 months, *Late MDA* MDA between 3 and 12 months, *Never MDA* no MDA within first year, *SF36* Short Form-36, *PCS* Physical Component Scale, *MCS* Mental Component Scale. All analyses corrected for baseline score, gender, age, symptom duration, use of disease-modifying antirheumatic drugs, erosive disease at baseline, and baseline disease activity (SJC, TJC, LEI, PASI, VAS global, VAS pain and HAQ)

### Productivity

At 1 year, 207 of the 250 patients who had completed productivity questionnaires (83%) were in the employable age. Of those, 153 (74%) were actually employed: 64 (87%) early MDA, 48 (76%) late MDA, and 41 (60%) never MDA patients (Table 3). Of the 53 unemployed subjects, 7 patients had become unemployed since diagnosis (1 early MDA, 3 late MDA, and 3 never MDA) and 46 were already unemployed at baseline. At 1 year, 8 reported long-term sick leave (1 late MDA (2%) and 7 never MDA (17%)) and 21 short-term sick leave in the past 4 weeks (10 early MDA (16%), 5 late MDA (11%), and 6 never MDA (18%)). Work productivity loss in the past 4 weeks was reported most often by never MDA patients (71%, vs. early MDA 27% and late MDA 30%). The mean productivity loss in patients with productivity loss did not differ significantly between the groups. Mean productivity at work of employed patients during the first year is shown in Fig. 1. Productivity increased in the late MDA group from a baseline level similar to that in the never MDA group, to a level similar to that in the early MDA group at 1-year follow-up. This was confirmed in the linear regression analysis (Table 2): total productivity at work was significantly lower at 1 year in the never MDA group than in the late MDA group (*β* − 7.38, 95% CI − 14.38, − 0.38). The difference in evolution over time between the three groups was confirmed in a linear mixed model analysis (LRT of interaction between time and group 6.93, *p* = 0.032).

**Table 3:** Productivity outcomes after 1 year in MDA groups and in total study population

	Early MDA (*n* = 91)	Late MDA (*n* = 73)	Never MDA (*n* = 86)
Employable age	74 (81)	64 (88)	69 (80)
Employed	64 (87)	48 (76)	41 (60)
Working hours per week	36 (33–39)	35 (31–38)	32 (28–37)
Long-term absence	0 (0)	1 (2)	7 (17)
Short-term absence	10 (16)	5 (11)	6 (18)
Productivity loss at work	17 (27)	14 (30)	24 (71)
Hours per week productivity loss at work in patients with productivity loss	6.7 (4–10.5)	7.6 (4.8–16.8)	7.9 (4.5–18.9)
Total productivity at work	32 (29–35)	29 (25–34)	19 (14–25)

Results shown as mean (95% confidence interval) or n (%) unless otherwise indicated. *Early MDA* MDA within 3 months, *Late MDA* MDA between 3 and 12, *Never MDA* no MDA within first year, *IQR* interquartile range. Total productivity = hours per week - absence - productivity loss at work, calculated in mean per week over past 4 weeks

### Radiological damage

Mean PsA-SHS was 3.5 (SD 10; 16 missing) at baseline: the erosion score was 1.4 (SD 4.6) and the narrowing score was 2.2 (SD 5.9). Mean progression in 1 year was 0.38 (SD 1.4; 31 missing). Both baseline and progression scores did not differ significantly between the groups. The PsA-SHS score had progressed in 34 patients (14%): 8 in the early MDA group (10%), 11 in the late MDA group (17%), and 15 in the never MDA group (17%). Twenty-seven of the 34 patients with progression in the first year had already radiological damage at baseline.

## Discussion

In this cohort of newly diagnosed PsA patients receiving usual care, HRQoL and productivity throughout the first year after diagnosis were best in those who reached MDA early. At 1 year, however, patients late in MDA had achieved similar levels of HRQoL and work productivity as patients in the early MDA group, even though they were significantly shorter in MDA. Patients never in MDA had significantly lower scores throughout the first year and this remained so at their 1-year visit. Radiological progression did not differ in the first year between the three groups.

In a previous cross-sectional analysis in our cohort, having reached a state of MDA was related to better HRQoL and functioning [11]. Other studies found similar results [10, 12]. The present study, however, is the first to address associations between gaining MDA and improvement of HRQoL and functioning over time. We found that gaining MDA, irrespective of the time elapsed since diagnosis, was associated with subsequent improvements of HRQoL and functioning over time. A slightly longer period of high disease activity was not associated with irreversible effects on HRQoL, functioning, or radiology after 1 year.

Still, one third of all patients was not able to achieve MDA in the first year and also did not show improvement in HRQoL and functioning. Even after correcting for baseline differences in disease activity and HRQoL scores, these patients’ SF36-PCS scores were 8 points lower than in the rest of the population. While patients in the late MDA group showed a clinically important improvement [19], patients in the never MDA group did not. Furthermore, almost all patients in the latter group reported some form of impact on their work: becoming unemployed, sick leave, or significant productivity loss at work. Thus, the impact of disease is severe and widespread in patients who do not achieve MDA. Interestingly, the baseline values of patients in the never MDA group did not differ much from those of patients in the late MDA group. If it were possible to predict achievement of MDA, patients at risk of not achieving MDA could be offered more intensive monitoring and earlier escalation of therapy.

Although analysis revealed a relation between time to MDA and HRQoL and productivity throughout the first year, a difference in radiological progression between the three groups was not shown. We assume that this is the consequence of a lack of power resulting from the strong association with damage present at baseline and the low prevalence of progression of 14%. Several causes for this low prevalence can be proposed. First, the 1-year follow-up was too short to demonstrate differences in this population. Second, radiological evaluation comprised assessment of hand and feet joints only, which are not necessarily the joints most often affected in for example oligoarthritis or monoarthritis patients. Third, disease activity in this usual care population at time of inclusion was lower than that in, for example, trial populations, for which higher progression rates have been reported [20, 21].

In this observational cohort, all patients with a new diagnosis of PsA were eligible to participate. They all received usual clinical care and their outcomes are therefore reflective of current clinical care. Their treatment differed, however, as treatment was not protocolized. This heterogeneity of treatment could be a confounder of the relation between time to MDA and outcomes. However, as choice of treatment was associated with disease activity, correcting for baseline disease activity and use of DMARDs in the analyses reduced a substantial part of the confounding. Further, correcting for time to starting DMARD therapy and time on DMARD therapy did not alter the effects of MDA group on the outcomes. Unmeasured confounding by treatment could be present but likely has not affected our conclusion, given the small differences in treatment between the three groups.

This study has several strengths and limitations. First, the population studied reflects the population of PsA patients receiving usual care. Thus, our conclusions are better generalizable than those from trials or biological registries, where patient selection bias might have occurred. Second, reliability of the clinical data collection is ensured because data were collected by research nurses in a standardized manner during dedicated study visits. As a limitation, this could however introduce discrepancies between disease activity and treatment strategy, as the latter is determined by physicians based on their own assessment of disease activity, unaware of the nurse’s assessment. We, therefore, cannot exclude incomplete correction for confounding by disease activity, although we also corrected for DMARD therapy. Another limitation is the heterogeneity of treatment strategies, as discussed above. There is a possibility of selective drop-out of patients. It could well be that patients with early MDA are discharged from clinical care and are less likely to complete follow-up and be included in our 1-year analysis. As a consequence, the rates of MDA and productivity may be underestimated, in which case the true effect of MDA on productivity is even larger. Last, in our analysis of data after 1 year, we were not able to show a difference between groups in MDA within 1 year on outcomes at 1 year, but we might see a difference in future analyses of patients later in MDA and with longer follow-up.

## Conclusion

In conclusion, patients who gained MDA in the first year after diagnosis showed considerable improvement in HRQoL and functioning, irrespective of the time elapsed to the first MDA episode. In one third of patients who did not gain MDA in the first year after diagnosis, the impact of disease remained as substantial as at the time of diagnosis.

## Abbreviations

DEPAR: Dutch southwest early psoriatic arthritis cohort
DMARDs: Disease-modifying antirheumatic drugs
HAQ: Health Assessment Questionnaire
HRQoL: Health-related quality of life
IQR: Interquartile range
LEI: Leeds Enthesitis Index
LRT: Likelihood ratio test
MDA: Minimal disease activity
PASI: Psoriasis Area and Severity Index
PCQ: Productivity Cost Questionnaire
PsA: Psoriatic arthritis
PsA-SHS: PsA-modified Sharp/van der Heijde score
SD: Standard deviation
SDD: Smallest detectable difference
SF36: Short Form 36
SF36-MCS: Short Form 36 Mental Component Scale
SF36-PCS: Short Form 36 Physical Component Scale
SJC: Swollen joint count
TJC: Tender joint count
VAS: Visual analog scale
